# Exploring the Mechanism of β-Cyclodextrin-Encased Phenolic Acids Functionalized with TPP for Antioxidant Activity and Targeting

**DOI:** 10.3390/antiox14040465

**Published:** 2025-04-13

**Authors:** Christopher Sbarbaro, Valeria Márquez-Miranda, Matías Leal, Ricardo Pino-Rios, Pedro Olivares, Makarena González, Ignacio Díaz-Franulic, Fernando González-Nilo, Osvaldo Yáñez, Yorley Duarte

**Affiliations:** 1 Center for Bioinformatics and Integrative Biology, Facultad de Ciencias de la Vida, Universidad Andrés Bello, Santiago 8370035, Chile; christopher.sbarbaro@usach.cl (C.S.); valeria.marquez@unab.cl (V.M.-M.); pedro.olivares@unab.cl (P.O.); m.gonzalezreyes2@uandresbello.edu (M.G.); ignacio.diaz@unab.cl (I.D.-F.); fernando.gonzalez@unab.cl (F.G.-N.); 2 Departamento de Química Orgánica y Fisicoquímica, Facultad de Ciencias Químicas y Farmacéuticas, Universidad de Chile, Santiago 8380494, Chile; matias.leal@ciq.uchile.cl; 3 Instituto de Ciencias Exactas y Naturales (ICEN), Universidad Arturo Prat, Playa Brava 3256, Iquique 1111346, Chile; rpinorios@unap.cl; 4 Centro de Modelación Ambiental y Dinámica de Sistemas (CEMADIS), Universidad de las Américas, Santiago 7500975, Chile; oyanez@udla.cl

**Keywords:** antioxidant, phenolic acids, complex, oxidative stress, triphenylphosphonium (TPP)-conjugated

## Abstract

Oxidative stress on the mitochondria in a human cell is attributed to several life-risking conditions, and as such, the importance of molecular structures packed with antioxidant properties and structural characteristics to enter the cell to help prevent such stress has been substantially relevant in recent years. In this study, we investigated the antioxidant properties of triphenylphosphonium (TPP)-conjugated phenolic acids encapsulated in β-cyclodextrin (β-CD). We synthesized TPP conjugates of caffeic, coumaric, and cinnamic acids and formed inclusion complexes with β-CD. Our results showed successful encapsulation of TPP conjugates in β-CD with high efficiency. The TPP conjugates maintained antioxidant activity, with slight reductions observed in β-CD complexes. Furthermore, cell viability studies showed low cytotoxicity of the dds. Computational analyses revealed that TPP conjugation preserved the chemical reactivity of the phenolic acids. Molecular dynamics simulations demonstrated stable inclusion complexes with β-CD and the free energy calculations indicated that TPP conjugation significantly enhanced the ability of caffeic acid to translocate across mitochondrial membranes. These results highlight the potential of TPP-conjugated phenolic acids encapsulated in β-CD as effective antioxidants with improved mitochondrial targeting capabilities.

## 1. Introduction

**Figure 1:**
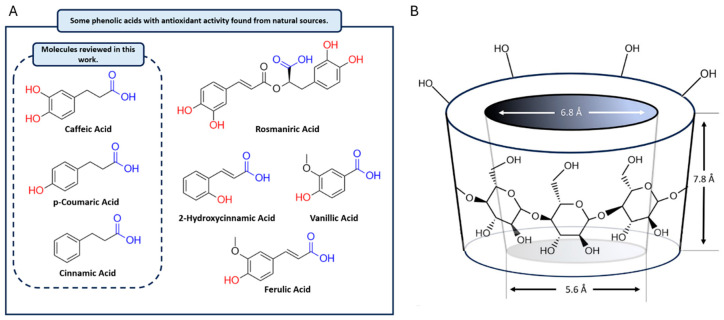
(**A**) Chemical structures of several phenolic antioxidants derived from natural sources. While the main focus is on caffeic acid, p-coumaric acid, and cinnamic acid, these compounds share several structural similarities, such as hydroxyl groups (red) and carboxylic groups (blue). (**B**) Structure of β-cyclodextrin (β-CD). The unique “cup” geometry of β-CD positions its hydroxyl groups in a way that creates a hydrophobic environment within the cavity. This allows the polyphenolic portion of the synthesized molecule, which has minimal steric and electrostatic repulsion, to be readily encapsulated within the β-CD cavity.

Diverse families of compounds have been derived from natural sources, given their potential pharmacological properties. Polyphenolic acids have been gathering attention thanks to their notable antioxidant properties (Figure 1A), due to their capacity to neutralize reactive oxygen species (ROS), a known factor in oxidative stress and damage within cells [1,2]. This kind of damage has been associated with the development of degenerative diseases, such as some types of cancer, cardiovascular diseases, and neurodegenerative disorders [3,4,5]. Recent studies have reinforced the significance of polyphenols in cancer prevention, highlighting their ability to induce cell cycle arrest and inhibit angiogenesis, although challenges such as poor bioavailability continue to hinder their clinical application [6]. There is more evidence pointing to these types of compounds for other potential applications, including their anti-inflammatory and anti-cancer effects. For instance, dietary polyphenols have shown promise in reducing cognitive side effects associated with cancer treatments and may lessen adverse effects of radiation therapy in breast cancer patients [7]. Despite the potential uses for these molecules, there are several challenges associated with therapeutic applications, such as their low solubility in water, and their tendency to degrade into derivatives with less activity when exposed to ultraviolet radiation [8]. In addition, for these compounds to exert their biological activity, there are several lipophilic barriers that they need to pass in order to reach their target sites, all while maintaining their structural integrity. Several strategies have been developed to overcome these limitations, with current formulations focusing on the use of nanomaterials [9], polymers [10], or polysaccharides [11], which have proved to protect their functionality, and in some cases improve them. Among these methods, the use of cyclodextrins (CDs) emerges as an attractive alternative due to their specific chemical and structural properties, being a good complement to polyphenolic structures (Figure 1B) [12].

CDs are cyclic oligosaccharides composed of glucopyranose units (Figure 1B). When pure, they are non-hygroscopic, homogenous crystalline structures whose conformation exposes a hydrophilic exterior and an internal cavity with hydrophobic properties. This amphiphilic quality gives CDs the ability to form inclusion complexes with molecules with hydrophobic structural segments, which is proven to improve their solubility and stability when they are in aqueous environments, even if their native solubility is poor [13,14]. Recent findings indicate that the encapsulation of polyphenols in CDs not only enhances solubility but also stabilizes these compounds against degradation during food processing and digestion [15]. The use of CDs has also allowed for improved means of administration, enabling oral, intravenous, transdermal and nasal delivery, making them an ideal choice for vehicles to overcome their limitations [16,17]. Chemical modification of naturally derived compounds to create novel molecules with enhanced targeting capabilities as an approach to improve pharmacokinetics and antioxidant activity has also been widely explored. In this context, the structural fragment that is known to improve mitochondria-targeting, (4-Cayboxybutyl)triphenylphosphonium (TPP) emerges as a viable alternative to facilitate the delivery of therapeutic agents to mitochondria, which is recognized as a critical site for oxidative stress related to ROS production [18,19].

To further improve the design and gain insight into potential mechanisms of action for novel compounds, quantum chemistry, and computational methods prove indispensable thanks to their robust modeling power and predictive potential. Studies involving the energy gap between HOMO and LUMO is a useful approach to predict molecular reactivity, identifying potential active sites within a structure and evaluating stability against oxidation in biological environments by assessing energy gaps within the electronic structure [20]. This strategy has also been applied to determine the stability of inclusion complexes, exploring the energies involved in the complexation and the filling of the CDs hydrophobic cavity [21]. Molecular dynamics (MD) simulations are also used to dig deeper into the mechanisms of complex formation and behavior, revealing that encapsulation not only improves solubility but also protects phenolic compounds from degradation during storage and digestion, thereby prolonging their antioxidant activity. These simulations provide valuable insights into the binding affinity and stability of inclusion complexes formed between TPP-modified phenylpropanoids and CDs [22]. By analyzing interaction energies and molecular trajectories, MD simulations help identify the most stable and energetically favorable complexes. Furthermore, MD simulations allow for the assessment of the affinity of these modified phenylpropanoids for biological membranes, such as cardiolipin-rich mitochondrial membranes. Recent findings suggest that the encapsulation process alters the physicochemical properties of phenolic acids, enhancing their durability and stability against light and thermal degradation. Free energy calculations derived from MD simulations quantify the strength of interactions, providing a robust framework for evaluating the targeting efficacy of these compounds [23].

In this study, TPP-modified phenylpropanoids were synthesized and evaluated for their potential as novel antioxidants. The experimental approach included assessing their antioxidant activity and conducting cell viability assays to ensure safety. Quantum chemistry calculations were performed to determine HOMO–LUMO energy gaps and molecular reactivity indices. These parameters provided a theoretical basis for understanding the electronic characteristics of the modified molecules. Additionally, MD simulations were conducted to investigate the stability and interaction energies of the TPP-modified phenylpropanoids with CDs and cardiolipin membranes. To evaluate the impact of TPP conjugation on the antioxidant properties and mitochondrial targeting of phenylpropanoids, HOMO–LUMO analyses were performed to assess their electronic properties related to ROS scavenging. Molecular dynamics (MD) simulations were conducted to examine the formation of inclusion complexes with CDs, as well as the interactions between the modified phenylpropanoids and mitochondrial membranes. These analyses aimed to provide insights into the solubility, stability, and potential mitochondrial localization of the modified molecules.

## 2. Materials and Methods

### 2.1. Materials

Antioxidants caffeic acid (3,4-dihydroxycinnamic acid, CAF), coumaric acid (4-hydroxycinnamic acid, COU), cinnamic acid (CIN), and ethylenediamine were purchased from Sigma, with a purity content of 99%. (4-carboxybutyl) triphenylphosphonium (TPP) as well as the catalysts 1-ethyl-3-(3-dimethylaminopropyl)carbodiimide (EDC) and N-hydroxysuccinimide (NHS) were purchased from Sigma. Native *β*-cyclodextrin (*β*-CD) was purchased from Merck, as were all solvents used for synthesis in this work. All chemical reagents used were of analytical grade. For HPLC analysis, mobile phase solvents methanol, acetonitrile, and water were purchased from Los Alquimistas (Santiago, Chile), and were HPLC grade.

**Scheme 1:**
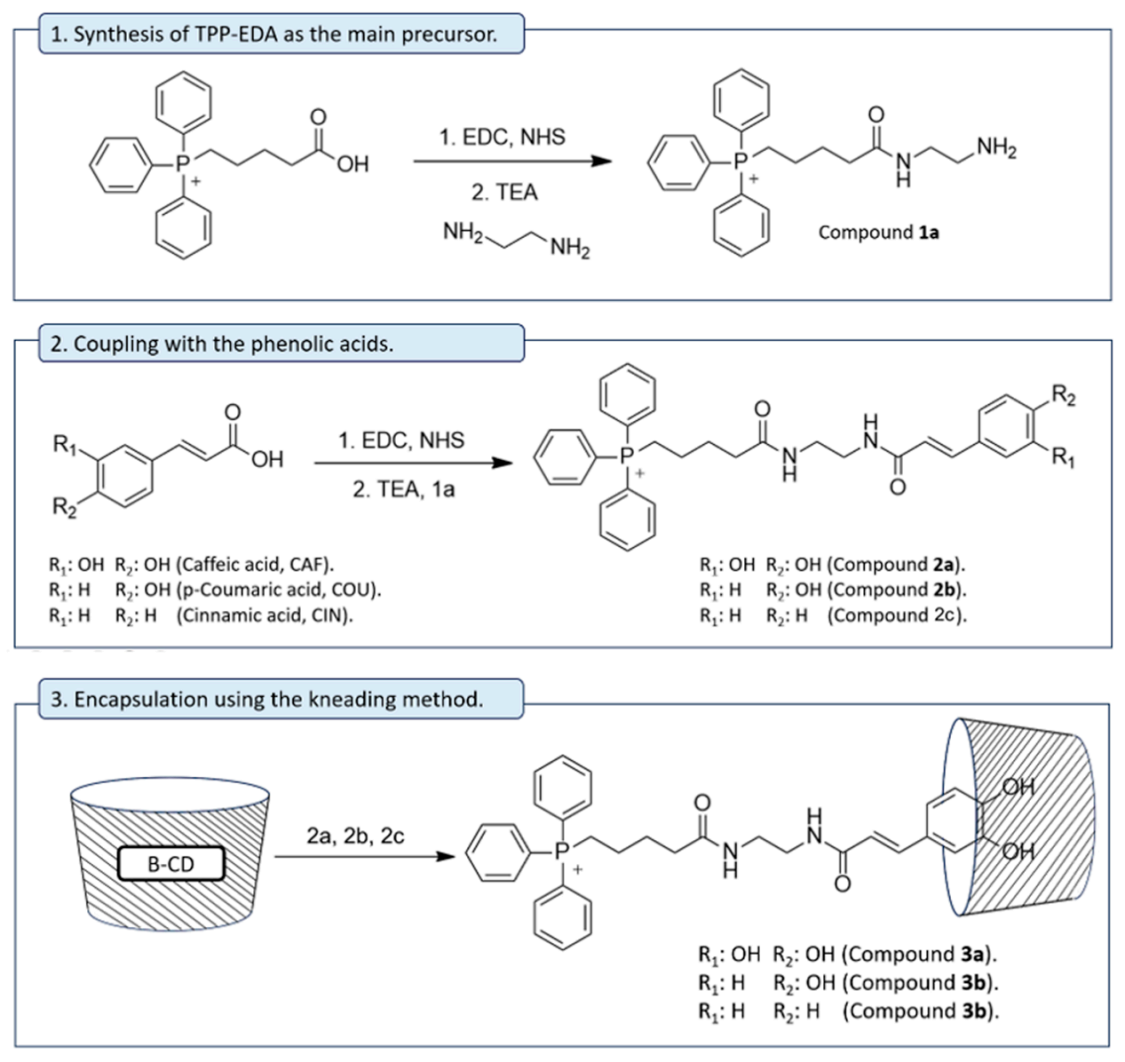
Schematic representation of the strategy used in this work, consisting of a three-step synthesis to chemically modify phenolic acids, followed by their encapsulation within a β-CD structure. The proposed structural arrangement positions the hydroxyl groups—key to radical scavenging mechanisms—inside the hydrophobic cavity of β-CD, enhancing stability and potential bioactivity.

Synthesis of TPP Antioxidant compounds (Scheme 1).

### 2.2. Synthesis of Compound ***1a***

For the coupling reaction, the selected phenolic acids (CAF, COU, and CIN) were activated before carrying out the amidation [24], for which 2.5 mmol of each one was dissolved with 0.432 g (3.75 mmol) of NHS and 0.583 g (3.75 mmol) of EDC in 5 mL of mixture consisting of MeOH/DCM (1:1) for 3 h at room temperature. Then, TPP was added together with 120 μL of TEA, and the reaction mixture was stirred for 24 h. The reaction crude mixture was concentrated using a rotatory evaporator and column chromatography using a hexane/ethyl acetate (6:3) mixture was used for purification. The fraction was isolated and dried to yield compound **1a**, which was verified through NMR spectroscopy before going forward.

### 2.3. General Method for TPP Antioxidant Synthesis (2a–c)

In three separate round-bottom flasks, each one for a different phenolic acid (CAF, COU, CIN), a mixture of EDC (3.7 mmol), NHS (3.7 mmol), and phenolic acid (3.09 mmol) was mixed in dry with nitrogen purge for 15 min. After that, 5 mL of DCM/MeOH 4:1 was added with constant stirring, and the mixture was allowed to react for 3 h at room temperature and inert atmosphere. To this flask, 3 mL of a solution consisting of the same solvent mixture, compound **1a** (3.09 mmol), and triethylamine (3.7 mmol) was added dropwise. The resulting mixture was allowed to react overnight. Contents of the flask were washed with water (3 × 15 mL) and brine (1 × 15 mL), and the isolated organic phase was dried with anhydrous NaSO_4_. The solvent was removed with rotatory evaporation and the oil was purified using flash column chromatography using hexane/ethyl acetate 6:3 solvent mixture as mobile phase.

### 2.4. Conjugation of TPP Antioxidant Within β-CD Matrix (3a–c)

Preparation of inclusion complexes was carried out using the kneading method [25]. Each inclusion complex (CAF/TPP, COU/TPP, and CIN/TPP) was weighted and poured separately into mortars containing β-CD so that the guest–host ratio is 1:1. Each mixture was kneaded by hand on a porcelain mortar with a pestle for 20 min using ethanol to keep a slurry consistency and adding more as it evaporated during the kneading process. The resulting product was then dried at 45 °C for 24 h and then the powder was ground further to obtain finer grains. Each product was stored at room temperature in a dry and light-protected vial.

### 2.5. Determination of Entrapment Efficiency

Remnant Antioxidant/TPP complex that did not interact with β-CD was obtained by washing the dry final compound with dichloromethane. The liquid residue was evaporated to obtain the solid, which was redissolved in HPLC quality solvent, after which it was filtered using 0.22 µm membrane. Quantification was carried out by high-performance liquid chromatography equipped with an Agilent UV-Vis detector (Agilent 1260 Infinity II HPLC, Santa Clara, CA, USA). Analysis of complexes were performed using a SunShell C18 column (150 mm × 4.60 mm i.d, pore size 2.6 µm) at 30 °C. Mobile phase was delivered through Agilent 1260 quaternary pump equipped with a degasser, maintaining an isocratic elution. The injection volume was 20 µL, and the column flow rate was 1.0 mL/min. UV was employed for detection at wavelengths set to 330, 325, and 310 nm for caffeic, coumaric, and cinnamic complexes, respectively. The results were compared using prepared calibration curves for each standard, prepared in a range of concentrations between 1 and 60 µM (fitting equations for caffeic, coumaric, and cinnamic acid derivates where y = 1819.2x − 5288.3, y = 1319.7x − 3665 and y = 760.51x − 639.6, with R^2^ values of 0.9975, 0.9936 and 0.9914, respectively), and entrapment efficiency was calculated using the following equation:

$$EE\left(\%\right)=\;\left(\frac{\left[Remnant\right]}{\left[Initial\right]}\right)\times \;100$$

where [Remnant] is the concentration of the obtained product through filtration and [Initial] is the amount of compound used for the kneading procedure.

### 2.6. ABTS and DPPH Scavenging Assays

The radical scavenging activity of antioxidants was evaluated using the ABTS and DPPH assays, which rely on the quenching of stable free radicals to assess antioxidant potential. The ABTS assay was conducted following the method outlined by Nenadis et al [shah and Modi, n.d] [26], using 2,2’-azinobis (3-ethylbenzothiazoline-6-sulfonic acid) (ABTS) to generate stable free radicals. A stock solution of ABTS (7 mM) was prepared in methanol, followed by the addition of potassium persulfate (2.45 mM) to produce the ABTS radical cation. The solution was stored in the dark at room temperature overnight. Prior to analysis, the ABTS/potassium persulfate solution was diluted with 80% methanol to achieve an absorbance of 0.7 at 734 nm. Prepared antioxidant solutions, synthesized in a 1:1 methanol–water mixture, were diluted to a concentration range of 0.01 to 0.5 mM. A volume of 16.1 µL of each antioxidant solution was mixed with 183.9 µL of ABTS solution. After 5 min of incubation, the absorbance of the samples was measured, using antioxidant-free ABTS solution as the control and pure methanol for blanking. The ABTS scavenging activity was calculated using the following equation:

$$ABTS\;Scavenging\;activity\;\left(\%\right)=\;\left(\frac{{Abs}_{A}-{Abs}_{B}}{{Abs}_{A}}\right)\;\times 100$$

where ${Abs}_{A}$ is the initial absorbance of the ABTS control (corrected with the blank), and ${Abs}_{B}$ is the absorbance of the reaction mixture. The DPPH assay was performed as described by Blois (1958) [27], employing 1,1-diphenyl-2-picryl-hydrazil (DPPH•) radicals. A freshly prepared 100 µM DPPH solution in methanol was diluted with 80% methanol until an absorbance of 0.8 at 517 nm was reached. Different concentrations of antioxidants and antioxidant complexes (0.01 to 0.5 mM) were mixed with the DPPH solution. After mixing, the samples were allowed to react for 30 min and were analyzed spectrophotometrically to monitor the shift from purple to colorless. Antioxidant-free DPPH solution served as the control, while a methanol–water mixture was used for blanking. The DPPH scavenging activity was determined using the following equation:

$$DPPH\;Scavenging\;effect\;\left(\%\right)=\;\left(\frac{{Abs}_{A}-{Abs}_{B}}{{Abs}_{A}}\right)\;\times 100$$

where ${Abs}_{A}$ is the initial absorbance of the DPPH control (corrected with the blank) and ${Abs}_{B}$ is the absorbance of the reaction mixture.

### 2.7. Cell Viability

Human cell lines used for viability studies, HEK293 and HeLa were provided by ATCC (Manassas, VA, USA). Both cell lines were kept at 37 °C in a humidified environment with 5% CO_2_ and cultivated in Dulbecco’s modified Eagle media supplemented with 10% fetal bovine serum, 100 units/mL of penicillin, and 100 mg/mL of streptomycin. A 100-μL aliquot of adherent cells was used to seed 96-well cell culture plates at a density of 25,000 cells/well. The cells were left to adhere for 16 h before the chemical was added. Each chemical was applied to cells for 48 h in DMEM with 10% FBS. After that, each well received 10 μL of the Cell Counting Kit-8 reagent (CCK-8; Sigma-Aldrich, Merck KGaA, Darmstadt, Germany), which was then incubated for 4 h at 37 °C in a humidified environment with 5% CO_2_. A Synergy H1 microplate reader (Biotek Instruments, Winooski, VT, USA) was used to detect absorbance at 450 nm in order to determine the vitality of the cells. Every experiment was carried out three times, with the statistical analysis being carried out with the software GraphPad Prism 10.4.1.

### 2.8. Quantum Chemical Calculations

Density functional theory (DFT) calculations for caffeic acid, *ρ*-coumaric acid, cinnamic acid, TPP, and compounds **2a**, **2b**, **2c**, selected for their antioxidant activity, were drawn using Discovery Studio [28]. Geometries were optimized using M06-2X functional [29,30] “comment” in conjunction with the 6-311++G(d,p) basis set, with Solvation Model Based on Density (SMD) for the implicit water, and the Grimme dispersion correction (D3). M06-2X-D3 is the best dispersion-corrected meta-GGA hybrid functional on the GMTKN30 database [31]. The Gaussian16 [32] software was used for all computations. Using the cubegen tool available in the Gaussian (v16) computational package, electron density and electrostatic potential of the molecules were first calculated in order to perform molecular electrostatic potential (MEP). The HOMO–LUMO gap, electronegativity (*χ*), global hardness (*η*), global electrophilicity index (*ω*), ionization potential (*I*), and electron affinity (*A*), and radical Fukui function (*f* ^0^), were the DFT-based global and local descriptors that were computed in order to gain better understanding of the molecules’ reactivity (Table 1). This method has been extensively used for the accurate determination of these properties in previous works [33,34,35,36].

The strategy for calculating the complexation energy scan of the complexes of *β*-CD, is as follows: each fragment of the system was considered separately, these fragments being the TPP antioxidants, *β*-CD, and the complex between TPP antioxidants and *β*-CD, each of which was optimized separately using the PM7 semi-empirical quantum mechanical method, with the implicit solvent model COSMO implemented in MOPAC16 [51]. We followed the method described by Guendouzi et al. [52] to locate the lowest energy minimum of TPP antioxidants@*β*-CD inclusion complexes. Two possible orientations of the TPP antioxidants in the complex were selected: Orientation A and B, which were mentioned above. The inclusion models can be seen on Figure 4b. The glycosidic oxygen atoms of the cyclodextrin molecule were placed onto the XY plane and their center was defined as the center of the coordination system. Then, TPP antioxidants were moved into the *β*-CD cavity along the Z-axis from −15.0 to +15.0 Å with 0.1 Å step. Subsequently, the heat of formation (Δ*H_f_*) was calculated for each fragment using PM7, with the implicit solvent model COSMO per each step. Finally, the complexation energy (*ΔE*) was obtained according to the following equation:

$$∆E=\;{∆H{}^{\circ}f}_{complex}-({∆H{}^{\circ}f}_{alcohols}+\;{∆H{}^{\circ}f}_{\beta -CD})$$

The non-covalent interaction index (NCI) [53,54] was used to qualitatively identify the areas where weak interactions predominate between TPP antioxidants@*β*-CD complexes, these interactions are dispersive in nature, hydrogen bonds, dipole–dipole interactions, or repulsive steric effects. In this work, the promolecular densities (*ρ*^pro^), computed as the sum of all atomic contributions, were used. The NCI was calculated using the NCIPLOT program (v4.2) [54]. The isosurfaces were visualized in Visual Molecular Dynamics (VMD) [55] (Humphrey et al., 1996).

The kinetic stability was evaluated using molecular dynamic simulations using the semiempirical tight-binding approach. All tight-binding molecular dynamics (MD) simulations were carried out using the GFN2-xTB Hamiltonian [56,57] with GBSA implicit solvation model of water. For each orientation (A and B, Figure 5) for Compounds **2a**, **2b**, and **2c** in the structure of *β*-CD inclusion complexes, 1.2 ns long production MD simulations were performed. During the MD simulations, the equations of motion were integrated with a 4.0 fs time step in the constant-number of atoms, constant-volume, and constant-temperature (NVT) ensemble at 300 K, using a Berendsen thermostat to maintain this constant temperature. The SHAKE algorithm was applied for all hydrogen atoms [58]. Data were collected every 100 fs during the MD runs. For molecular visualization of the systems and MD trajectory analysis the VMD software package (v1.9.4) was used.

### 2.9. Free Energy Calculations of TPP Conjugates Across Membranes

The free energy profile or potential of mean force for TPP–caffeic acid conjugate and unconjugated caffeic acid crossing a mitochondrial-like membrane was estimated by means the umbrella sampling method [59]. To this end, a 130 × 130 × 50 Å^3^ membrane patch composed by X lipids was built and a 30 × 130 × 130 Å^3^ water layer was added to the two sides of the z axis. Two systems were considered using either a single TPP–caffeic acid or caffeic acid molecules. The molecule was placed in the upper side of the system and restrained using a harmonic restrain of 10 kcal/mol × Å^2^ to run an equilibration by 100 ns.

The reference temperature and pressure were set at 310 K and 1 atm, respectively. The Nose–Hoover method was used to couple the temperature, and a semi-isotropic pressure scheme was used to maintain the overall pressure. Time constants of 0.1 and 1.0 ps were taken into consideration for temperature and pressure, respectively. The Particle Mesh Ewald (PME) method was used to calculate the long-range Coulomb interactions. 1.4 nm was chosen as the cut-off distance for van der Waals and Coulomb interactions. Two fs was set as the timestep.

The reaction coordinate for the umbrella sampling approach was specified as the distance, projected in the Z axis, between the molecule’s center of mass and the membrane’s center of mass to replicate the translocation of the molecule across the membrane. There were 56 1 Å windows created from the reaction coordinate. For every window, the harmonic spring’s force constant was 1 kcal/mol × Å^2^. Each window was run for at least 10 ns, giving each system a simulation time of roughly 600 ns. After that, the weighted histogram analysis technique (WHAM) [60] was used to compute PMF profiles. The AMBER package [61] was used to execute all the simulations.

Cardiolipins (TLCL lipids) were modeled using the CHARMM36 force field [62]. The caffeic acid was parameterized using CHARMM General force field (CGenff) [63]. TPP–caffeic acid force field parameters were obtained using the Force Field Toolkit plugin [64] in VMD software v1.9.4 [55].

## 3. Results and Discussion

### 3.1. Chemistry

Synthesized molecules were characterized by NMR spectroscopy, where integration of the signals confirmed the presence of the proposed structures of target molecules present in Table 2.

^a^. Pale yellow solid; yield: 82%; 1H NMR (400 MHz, MeOD) δ 7.97–7.88 (m, 5H), 7.87 (s, 2H), 3.38 (s, 5H), 3.26 (q, J = 7.3 Hz, 1H), 2.30 (t, J = 7.2 Hz, 3H), 1.88 (p, J = 7.3 Hz, 4H), 1.80–1.66 (m, 4H), 1.36 (t, J = 7.3 Hz, 2H).

^b^. Orange solid; yield: 67%; IR (KBr)) ν/cm^−1^ = 780, 836, 1009, 1078, 1176, 1247, 1439, 1527, 1699, 2955; 1H NMR (400 MHz, MeOD) δ 7.57 (d, J = 15.9 Hz, 1H), 7.10–7.02 (m, 2H), 6.97 (dd, J = 8.3, 2.1 Hz, 1H), 6.81 (d, J = 8.1 Hz, 1H), 6.29 (d, J = 15.9 Hz, 1H), 3.79 (s, 4H), 3.70 (s, 6H), 3.65 (s, 6H), 3.39 (t, J = 6.7 Hz, 5H), 2.55 (t, J = 6.7 Hz, 5H); 13C NMR (101 MHz, MeOH-d4) δ 174.10, 169.96, 149.79, 147.14, 147.03, 127.90, 123.12, 116.70, 115.33, 115.05, 40.38, 37.94, 35.44.

^c^. Pale yellow solid; yield: 53%; IR (KBr); 1H NMR (400 MHz, MeOD) δ 7.86–7.73 (m, 4H), 7.63 (d, J = 16.0 Hz, 2H), 7.50–7.44 (m, 4H), 6.87–6.79 (m, 4H), 6.34 (d, J = 15.9 Hz, 2H), 3.78 (s, 5H), 3.25 (t, J = 6.4 Hz, 6H), 1.97–1.80 (m, 5H); 13C NMR (101 MHz, MeOH-d4) δ 169.06, 160.62, 145.88, 130.84, 126.48, 116.19, 114.29, 35.26, 26.38, 25.63.

^d^. Pale yellow solid; yield: 51%; IR (KBr) ν/cm^−1^ = 780, 1010, 1174, 1258, 1438, 1517, 1694, 2954; 1H NMR (400 MHz, MeOD) δ 7.82–7.73 (m, 1H), 7.64–7.47 (m, 6H), 7.47–7.30 (m, 6H), 6.72–6.48 (m, 2H), 3.74–3.57 (m, 4H), 3.50–3.34 (m, 4H), 3.33–3.26 (m, 3H), 2.75–2.47 (m, 6H), 2.41 (td, J = 7.1, 4.6 Hz, 1H); 13C NMR (101 MHz, MeOH-d4) δ 168.30, 142.07, 135.62, 130.19, 128.21, 128.18, 128.06, 121.19, 36.23, 35.07, 25.54.

### 3.2. Entrapment Efficiency and Antioxidant Activity

The Kneading method was used for the formation of guest–host complexes between the synthetized antioxidants and b-CD structures as the use of ethanol allowed for an increased solubility of the guest molecules, favoring the interaction with the b-CD as the solubility of the original antioxidants is known to not be favorable in aqueous media. Different percentages of entrapment efficiency can be related to the difference in size in the aromatic section of the molecule that coordinates with the inner cavity in the b-CD structure, where up to 20.6% variation can be seen between the different complexes. Among the tested compounds, the TPP–Caf complex exhibited the highest encapsulation efficiency at 67.8%, followed by the TPP–Cou complex with 62.8%. The TPP–Cin complex showed the lowest encapsulation efficiency, reaching 47.2%. These results suggest that the nature of the antioxidant influences the interaction with β-CD, potentially due to structural differences affecting host-guest complex formation.

The scavenging activity of both ABTS and DPPH radicals increased proportionally with the concentration of antioxidant compounds (Table 3 and Table 4). However, a slight reduction in scavenging activity was observed when antioxidants were present as β-CD host–guest complexes. This decrease may be attributed to the structural orientation of β-CD, which coordinates around hydroxyl groups essential for interacting with reactive oxygen species (ROS). Antioxidants derived from caffeic and coumaric acids demonstrated higher scavenging activities compared to cinnamic acid derivatives, likely due to the presence of additional hydroxyl groups capable of enhancing ROS interactions. The concentration and form of guest material, whether as a host or a guest, affected the antioxidant activity observed. This means that the scavenging activity of both DPPH and ABTS radicals increased in proportion to the concentration of antioxidant compounds (Table 4 and Table 3), yet the scavenging effect had an overall tendency to slightly drop when the antioxidant was in the presence of β-CD as guest–host complex. Additionally, these compounds based on caffeic and coumaric acid as main antioxidants were found to be more effective than the ones derived from cinnamic acid. This can be associated with the presence or lack of hydroxyl groups capable of interacting with ROS as a main mechanism for their antioxidant activity. This could also explain how the scavenging effect drops when in the presence of β-CD, as the structural disposal is such that the host coordinates around the ring where hydroxyl groups are present, decreasing the chances of said groups to further interact with radical species present in the solution.

### 3.3. Cell Viability Studies

**Figure 2:**
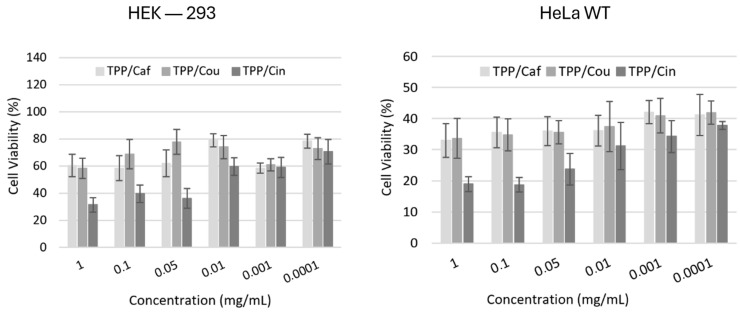
Effect of the selected compounds on cell viability using the CCK-8 assay in HeLa (**right**) and HEK-293 (**left**) cell lines. Concentrations used were 1 mg/mL, 0.1 mg/mL, 0.05 mg/mL, 0.001 mg/mL, and 0.0001 mg/mL. All values for error bars are means ± SD (n = 3). SD: standard deviation.

HEK-293 and HeLa WT cells were treated with a range of concentrations of the encapsulated antioxidants, as presented in Figure 2. For HEK-293 cells, a dose-dependent decrease in viability was observed across all treatment samples, with the peak in cytotoxic effect occurring in 1 mg/mL and 0.1 mg/mL. Among these encapsulates, coumaric acid derivate (TPP/Cou) exhibited the most pronounced cytotoxic effect, notably on the higher concentration range. A partial recovery in viability was observed in the range of 0.01 mg/mL of encapsulate, suggesting a threshold effect beyond, in which the cytotoxic effect is maximized. Overall observer tendencies were observed to be similar in HeLa WT cells, with the maximum viability being 40% approximately, even on the lowest concentrations. A dose-dependent trend was also observed, with a lesser impact over cell viability compared to HEK-293. Among the encapsulates used, TPP/Caf was the one that presented a milder effect in HeLa cells, whereas the other two encapsulates, TPP/Cou and TPP/Cin, caused a bigger impact towards cell viability. Differences between cell lines used can be attributed to variations in metabolic activity, permeability of the membranes or specific interactions.

### 3.4. Molecular Reactivity Analysis

Several chemical descriptors can be computed using Koopman’s approximation, which is based on the frontier molecular orbital theory. These descriptors, which can be classified as either global or local, are helpful in forecasting the reactivity of both atoms and molecular complexes. While the latter enables the investigation of individual sections within a molecular species, the former is especially helpful for analyzing the reactivity of a molecule in its entirety. In addition to the radical Fukui function, the most commonly employed descriptors are likely ionization energy (I), electron affinity (A), electronegativity (χ), global hardness (η), and global electrophilicity index (ω).

Table 5 presents the global chemical reactivity descriptors for caffeic acid, *ρ*-coumaric acid, cinnamic acid, Compound **2**, Compound **3**, Compound **4**, and TPP using the Koopman’s approach. We have included the ionization potentials in the results as a simple descriptor to indicate the feasibility of possible reactions that involve electron transfer prior to the proton transfer to a potentially free radical-derived base. Considering this simple model, the values of the *I* between the analyzed molecules are similar with the caffeic acid–Compound **2**, *ρ*-coumaric acid–Compound **3**, and cinnamic acid–Compound 4. Therefore, the combination of TPP with the caffeic acid, *ρ*-coumaric acid, and cinnamic acid antioxidants, keep similar *I* values when formed as Compounds **2**–**4**. This same behavior is reflected when considering the values for *η* and *χ*, where *η*, indicating that they are less averse to electron arrival and are thus more reactive systems and *χ*, indicates the ability to attract electrons. The ***ω*** values are also similar between antioxidants caffeic acid, ρ-coumaric acid, and cinnamic acid with Compounds **2**–**4**, this shows that Compounds **2**–**4** are highly stable in terms of electron acceptance and with a similar reactivity to the initial antioxidants.

**Figure 3:**
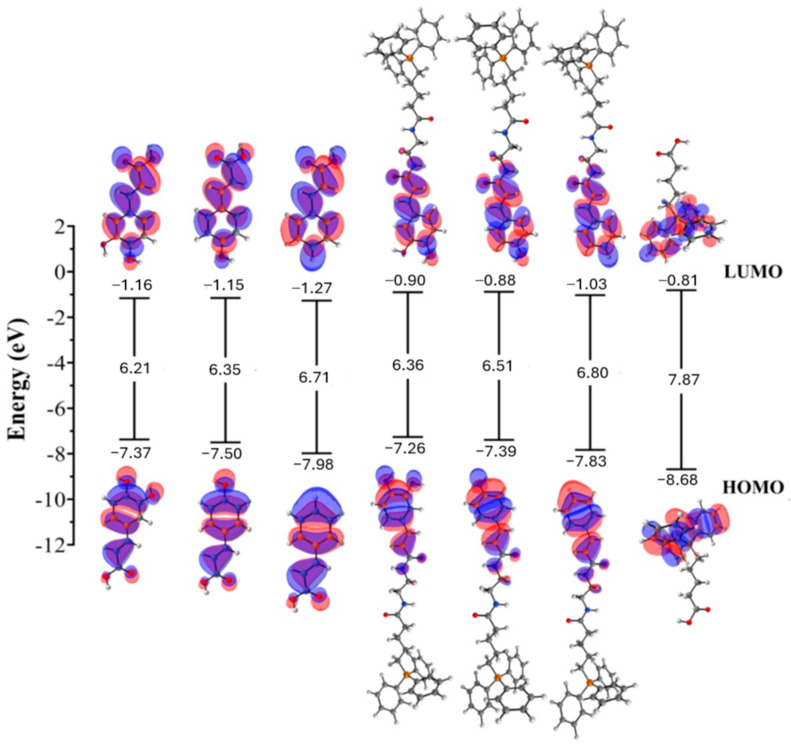
Frontier molecular orbitals and HOMO–LUMO energy gap of caffeic acid, *ρ*-coumaric acid, cinnamic acid, Compound **2**, Compound **3**, Compound **4**, and TPP at the M06-2X-D3/6-311++G(d,p) level of theory.

For highest occupied molecular orbital (HOMO) and the lowest unoccupied molecular orbitals (LUMO) energies and their difference. The HOMO–LUMO isosurfaces, shown in Figure 3, follow the same pattern of lobes between caffeic acid–Compound **2**, *ρ*-coumaric acid–Compound **3**, and cinnamic acid–Compound **4**, clearly shows that the chemical reactivity of the antioxidants is maintained in Compounds **2**–**4**. When we consider the energy gap values, the difference between caffeic acid–Compound **2** is 0.15 eV, for *ρ*-coumaric acid–Compound **3** it is 0.16 eV, and cinnamic acid–Compound **4** it is 0.09 eV. Therefore, the energy gap is similar between the antioxidants and Compounds **2**–**4**, maintaining the reactivity of the molecules.

The Molecular Electrostatic Potential maps (MEP) have been widely used as an indicator of molecular reactivity, as the most negative values correspond to the regions of the molecule that are most susceptible to electrophilic attack. MEPs have been widely used as an indicator of molecular reactivity, as the most negative values correspond to the regions of the molecule that are most susceptible to electrophilic attack. Mapping the electrostatic potential along the geometry of the molecule, its most reactive sites can be observed by means of a color code that extends across the structure, with red showing the most negative values (where there is the highest contribution of electrons to the electrostatic potential) and blue showing the most positive values (where the contribution of nuclei is highest). As shown in Supplementary Figure S3, the MEP reveals a coincidence between the most reactive sites for caffeic acid–Compound **2** and *ρ*-coumaric acid–Compound **3**, in the phenolic group, and for cinnamic acid–Compound **4**, in the phenyl group. The MEPs of molecules showed negative zones (red color) of electrostatic potential in the position of the hydroxyl groups, which are favored for electrophilic attacks, and the aromatic rings presented a slight negative zone, which can facilitate π–π stacking interactions. Thus, the attacking electrophile would be attracted to the negatively charged sites. These similarities in the reactive sites, potentials and charges around the compounds may be mainly attributable to the antioxidant activity for Compounds **2**–**4**. MEPs is especially useful for determining the reactivity of sites that exhibit non-covalent interactions with the other reactive species, i.e., hard–hard interactions dominated by electrostatic effects [65,66].

After analysis of the results obtained by the MEP, the results of the radical Fukui function (*f* ^0^) were mapped, see Supplementary Figures S3 and S4. The chemical interpretation of *f* ^0^ is that higher isovalues of this descriptor represent the regions in a molecule that are most susceptible to free radical attack. Therefore, it is known that free radicals and antioxidants in nutrition, health, and human pathology, antioxidants are responsible for the removal of such radical species [67]. According to the local hard–soft acid base (HSAB) principle, the regions where the Fukui function is highest are chemically softer regions [68], and, as can be seen in Supplementary Figures S3 and S4, these reactive sites coincide for caffeic acid–Compound **2** and *ρ*-coumaric acid–Compound **3**, in the phenolic group, and for cinnamic acid–Compound 4, in the phenyl group. These sites (isosurfaces) show a marked similarity in the softness (and thereby the reactivity) of each of the double bonds of each of the structures and hydroxyl groups studied. Therefore, the radical Fukui function, in contrast to the MEP, was able to show in greater detail the similarities between the reactive sites of caffeic acid–Compound **2**, *ρ*-coumaric acid–Compound **3**, and cinnamic acid–Compound 4. These results indicate that the combination of TTP and the antioxidants caffeic acid, *ρ*-coumaric acid, and cinnamic acid, to form Compounds **2**–**4**, are equally probable to occur at the reactive sites described above. The results obtained are in accordance with the experimental data reported in this work.

### 3.5. Complexation Energies and Kinetic Stability

**Figure 4:**
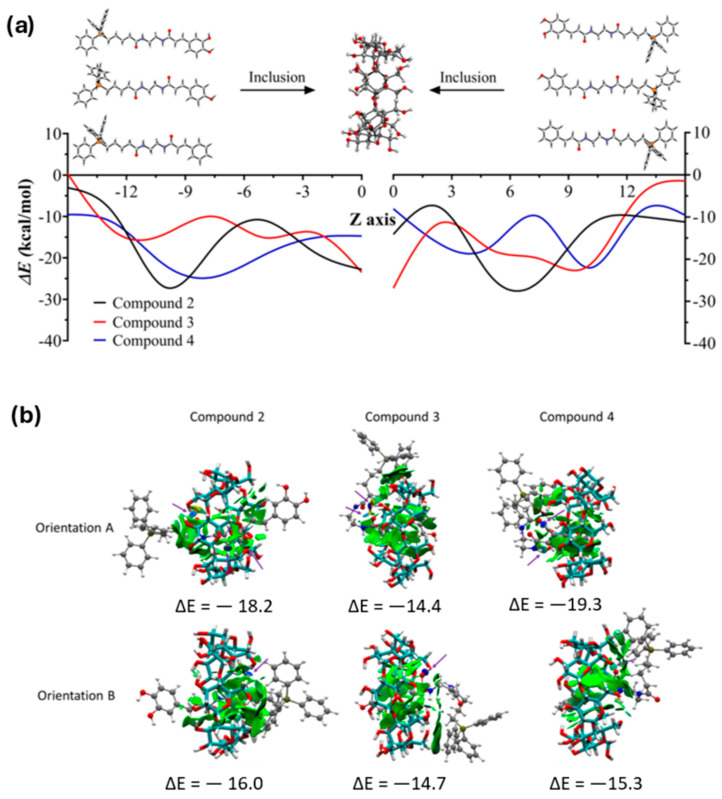
(**a**) The coordinate system used to define the process of complexation of TPP antioxidants in *β*-CD. Orientations A (head up) and B (head down) in *β*-CD cone in the 1:1 complexes. The relationship between the energy and the position (axis Z) of the each stepwise for TPP antioxidants in at PM7 level of theory. (**b**) Complexation energy (ΔE) and NCI isosurface gradient (0.8 au) of different orientations (A and B) for TPP antioxidants in the structure *β*-CD inclusion complexes at PM7 level of theory. The surfaces are colored on a blue–green–red scale according to the strength and type of interaction. The purple arrows indicate H-bonds.

The insertion of Compounds **2**–**4** into the *β*-CD cavity was investigated by semi-empirical calculations with the PM7 method, where a scan of the surface potential was performed in a fast and efficient way, two orientations A and B (Figure 4b) were considered for potential energy profiling. The results of the profiles show that for orientation A, Compounds **2**–**4** are more energetically favored than for orientation B. The change in the complexation energy to much more negative values indicates a more favorable inclusion. Thus, orientation A is more energetically favored with an energy difference between orientation B of 3.1 kcal/mol, indicating therefore a favorable thermodynamic process, see Figure 4. The average complexation energy values for orientation A are as follows: Compound **2** is −18.2 kcal/mol; Compound **3** is −14.4 kcal/mol; and Compound **4** is −19.3 kcal/mol. For orientation B, the average values of complexation energy are as follows: Compound **2** is −16.0 kcal/mol; Compound **3** is −14.7 kcal/mol; and Compound **4** is −15.3 kcal/mol.

β-CDs can create water-soluble complexes with water-insoluble lipophilic substances by forming hydrogen bonds with other molecules through the hydroxyl groups on their exterior surface. Non-covalent interaction (NCI) characterization is crucial for molecular recognition systems. Through isosurface mapping, we have used NCI analysis to comprehend the type of intermolecular interactions that take place in TPP antioxidants@β-CD complexes. Figure 4 displays NCI plots for each molecule, which were created using an isovalue of 0.6 a.u. Strong attractive interactions are represented by the color blue, whereas weak van der Waals interactions are represented by the color green. As can be seen in Figure 4b, the isosurfaces show that the hydroxyl groups of β-CD and the C=O and N-H atoms of Compounds **2**–**4** have weak hydrogen bonds (blue disks) and van der Waals interactions (green colored fields). Intermolecular hydrogen bonding [69] and weak van der Waals interactions both contribute to the development of TPP antioxidants@β-CD complexes.

To show the kinetic stability of the TPP antioxidants in the structure *β*-CD inclusion complexes on different orientations (A and B), tight-binding molecular dynamics simulations were carried out with a time duration of 1.2 ns, where the root mean square deviation (RMSD) over time has been evaluated (Figure 4a). The results indicate that the TPP antioxidants in the structure *β*-CD inclusion complexes are highly stable kinetically, which is in agreement with the experimental results. For the case of the systems under study, the trend is highly stable, since the promising RMSD values fluctuating below 2 Å is quite good according to Li and Col. [70]. For this reason, it is possible to state that the three-dimensional complexes are conserved over time, indicating good kinetic stability. Our molecular dynamics simulations also revealed that while the hydroxyl groups remain partially accessible in the complex, the approach angle for free radicals becomes restricted, increasing the activation energy required for hydrogen atom transfer. This phenomenon is supported by recent work carried out on similar systems using tight binding approaches [71,72,73].

The non-covalent interaction (NCI) analysis we performed further supports this mechanism, showing weak hydrogen bonds and van der Waals interactions between the hydroxyl groups of our compounds and the interior of the β-CD cavity, which would alter the electronic environment of these groups and consequently their radical scavenging capacity.

### 3.6. Interaction of TPP Conjugates with Mitochondria Model Membranes

Once the cyclodextrin–TPP-antioxidant complexes have been uptaken by the cells, it is expected that the TPP–antioxidant molecule be released into the cytoplasm, where TPP may guide its traffic to the mitochondria. At the molecular level, the inner mitochondrial membrane demonstrates a distinctive composition characterized by the virtual absence of cholesterol and the prevalence of cardiolipin (CL). Cardiolipin is a unique anionic phospholipid predominantly found in mitochondrial membranes, where it plays a crucial role in various mitochondrial functions such as metabolism, membrane dynamics, and apoptosis. The specific concentration of cardiolipin within mitochondrial membranes impacts membrane structure and dynamics [74]. Thus, the presence of cardiolipins makes the mitochondrial inner membrane different from the other cell membranes. Accordingly, to investigate whether the conjugation of TPP to caffeic acid enhances its ability to translocate across mitochondrial membranes, we performed calculations to estimate the potential of mean force (PMF) profiles of both molecules (TPP–caffeic acid and caffeic acid) across a cardiolipin membrane as a model system. Considering this, two systems were built (as described in Materials and Methods) that incorporated these lipid components as previously reported [75]. The umbrella sampling method was employed to estimate PMF.

**Figure 5:**
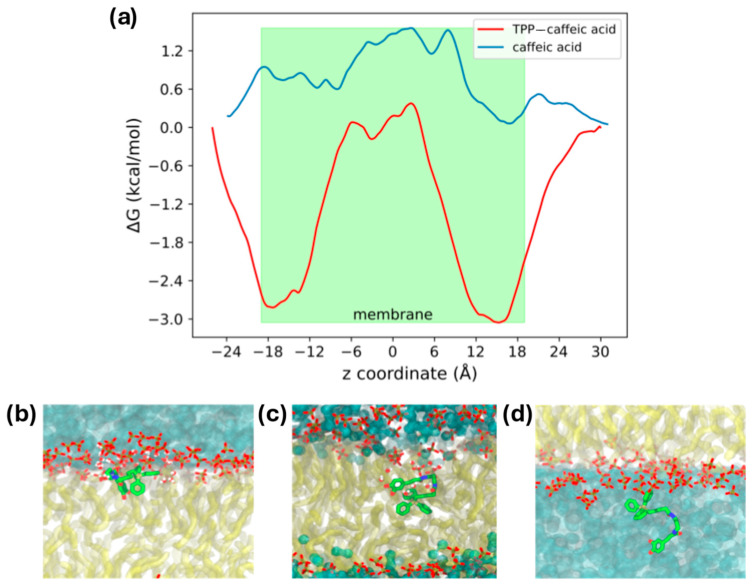
(**a**) Free energy profiles of the translocation of caffeic acid and TPP–caffeic acid conjugate through inner mitochondria model membrane. The profiles were obtained using the Umbrella Sampling method. (**b**–**d**) are snapshots of the TPP–caffeic acid conjugate at (**b**) z = 15, the water–membrane interface; (**c**) z = 0, the center of the membrane; and (**d**) z = −24 the water environment.

Figure 5b illustrates that unconjugated caffeic acid exhibits limited membrane translocation possibilities, as positive free energies are observed throughout the molecule’s passage across the membrane, with a higher barrier towards the center. Conversely, in the case of TPP-conjugated caffeic acid, a minimum at z = 15 Å, just below the membrane–water interface, is observed from right to left in Figure 5b–d. In this region, TPP-caffeic acid backfolds, and the rings tend to favor interaction with the membrane lipids, while the positively charged group of the delocalized cation exhibits a favorable interaction with the membrane’s phosphate groups. The molecule then overcomes an energy barrier of 3 kcal/mol to reach the center of the membrane.

Then, the molecule overcomes an energy barrier of 3 kcal/mol to reach the center of the membrane (z = 0 Å), where as depicted in Figure 5b, the TPP–caffeic acid drags some water molecules and slightly disorganizes the lipids in that area, since the caffeic acid moiety gives polar characteristics to the molecule. As expected, in the lower leaflet of the membrane, the molecule encounters another minimum of approximately −3 kcal/mol before transitioning towards the polar heads of the membrane (z = −15 Å). Subsequently, another energy barrier is observed as it exits towards the solvent (z = −24 Å), where the molecule unfolds and TPP moiety interacts by the last time with the phosphate groups of the membrane, while the caffeic acid section stretches towards the water.

These findings demonstrate that TPP facilitates the translocation of the caffeic acid molecule to inner mitochondrial membranes, with a higher affinity for the membrane–water interface. This reinforces the notion that TPP holds excellent potential for incorporation into delivery systems aimed at directing compounds towards the mitochondria.

## 4. Conclusions

Based on the findings of this study, it can be concluded that the kneading method can effectively form guest–host complexes between synthetized antioxidants based around phenolic compounds and *β*-CD structures. The use of ethanol was found to give appropriate solubility of the guest molecules, thereby facilitating their interaction with the *β*-CD. The size of the aromatic section of the molecule was found to affect the entrapment efficiency of the complexes, with up to 20.6% variation observed between different complexes. The concentration and form of the guest material were found to influence the antioxidant activity observed, with an overall tendency for the scavenging effect to slightly drop when the antioxidant was in the presence of *β*-CD as a guest–host complex. Compounds based on caffeic and coumaric acid were found to be more effective than those derived from cinnamic acid, where the activity seems to be proportional to the number of hydroxyl groups that can interact with ROS as a main mechanism for their antioxidant activity. The study demonstrates that combining TPP with caffeic acid, ρ-coumaric acid, and cinnamic acid to form Compounds **2**–**4** preserves their chemical reactivity and stability, as evidenced by similar global descriptors and HOMO–LUMO energy gaps. Molecular Electrostatic Potential (MEP) and Fukui function analyses identified consistent reactive sites in the phenolic and phenyl groups, supporting their antioxidant potential. Additionally, Compounds **2**–**4** formed stable inclusion complexes with β-cyclodextrin (β-CD), with orientation A being thermodynamically favored. Non-covalent interaction analysis revealed weak van der Waals forces and hydrogen bonds contributing to complex stability, further confirmed by molecular dynamics simulations showing high kinetic stability.

Finally, using free energy calculations, we demonstrate that conjugating TPP to caffeic acid significantly enhances its ability to translocate across inner mitochondrial membranes. While unconjugated caffeic acid faces a high energy barrier for translocation, the presence of TPP facilitates its passage through the membrane, favoring interactions with membrane phosphates and slightly reorganizing lipids. These findings highlight the potential of TPP as an efficient chemical group for directing compounds to the mitochondria, which could be valuable for drug delivery strategies. The results obtained from the various descriptors used in this study are in accordance with the experimental data reported, indicating the effectiveness of these tools in predicting molecular reactivity.

## Figures and Tables

**Table 1:** Equations for global and local reactivity indexes calculated in TAFF pipeline [37].

	Koopmans’ Theorem	Reference
Electronegativity (*χ)*	$\chi =-\frac{1}{2}{\left(\epsilon_{LUMO}+\epsilon_{HOMO}\right)}^{*}$	[38,39,40]
Electrophilicity (*ω*)	$\omega =\frac{\mu^{2}}{2\eta }=\frac{{\left(\epsilon_{LUMO}+\epsilon_{HOMO}\right)}^{2}}{2(\epsilon_{LUMO}-\epsilon_{HOMO})}$	[41]
Global hardness (*η*)	$\eta =\frac{1}{2}\left(\epsilon_{L}-\epsilon_{H}\right)$	[38,42,43,44,45,46]
Ionization potential (*I*)	$I=-\epsilon_{HOMO}$	[37]
Electron Donator (*A*)	$A=-\epsilon_{LUMO}$	[37]
HOMO–LUMO Gap (Δ*E_gap_*)	${\Delta E}_{gap}=\epsilon_{LUMO}-\epsilon_{HOMO}$	[37]
Radical Fukui function (*f* ^0^)	$f^{0}\left(r\right)=\frac{{\left|\phi_{L}(r)\right|}^{2}-{\left|\phi_{H}(r)\right|}^{2}}{2}$	[47,48,49,50]

* $\epsilon_{LUMO}$ is the lowest unoccupied molecular orbital (LUMO) energy, and $\epsilon_{HOMO}$ is the highest occupied molecular orbital (HOMO) energy. $\phi_{L}(r)$ is LUMO density, and $\phi_{H}(r)$ is HOMO density.

**Table 2:** Structures and NMR characterization of synthetized molecules.

n°	IUPAC Name	Molar Mass (g/mol)	Structure
1	(5−((aminoethyl)amino) −5−oxopentyl)triphenylphosphate(III) bromide ^a^	485.40	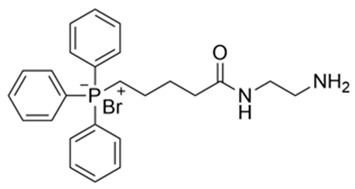
2	(E)−(5−((2−(3−(3,4−dihydroxyphenyl)acrylamido)ethyl)amino)-5-oxopentyl)triphenylphosphate(III) bormide ^b^	647.54	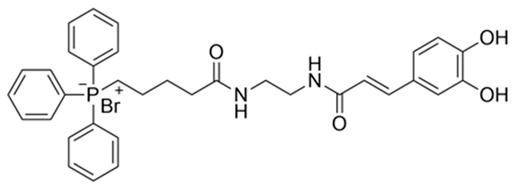
3	(E)−(5−((2−(3−(4−hydroxyphenyl)acrylamido)ehtyl)amino) −5−oxopentyl)triphenylphosphate(III) bormide ^c^	631.54	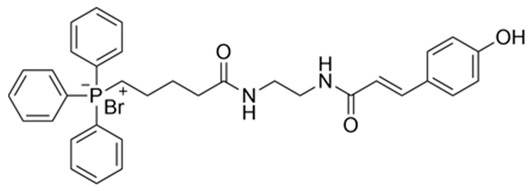
4	(E)−(5−((2−cinnamamidoethyl)amino)-5-oxopentyl)triphenylphosphate(III) bormide ^d^	615.54	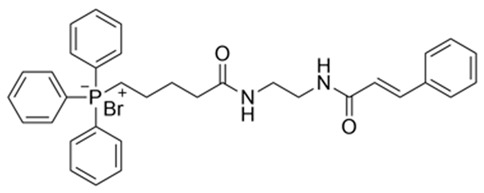

**Table 3:** Results for ABTS scavenging assay, comparing the antioxidant on its own, conjugated with 4-TPP, and the inclusion complex with β-cyclodextrin.

ABTS Radical Scavenging Activity (%)					
Sample Concentration (mM)					
Sample Name	0.01	0.05	0.1	0.25	0.5
CAF	66.5 ± 1.8	≥99	≥99	≥99	≥99
COU	19.0 ± 0.5	34.6 ± 1.4	61.3 ± 2.0	71.7 ± 8.1	99.7 ± 0.3
CIN	10.6 ± 0.7	8.9 ± 1.7	9.9 ± 3.3	10.2 ± 2.3	11.4 ± 1.9
4-TPP/CAF	49.1 ± 2.7	99.7 ± 0.3	≥99	≥99	≥99
4-TPP/COU	8.2 ± 2.4	16.4 ± 1.4	24.7 ± 1.3	48.8 ± 7.9	73.6 ± 11.1
4-TPP/CIN	13.1 ± 1.9	14.3 ± 0.7	27.88 ± 4.7	35.7 ± 1.9	44.5 ± 5.3
4-TPP/CAF/B-CD	34.5 ± 1.0	98.7 ± 0.5	≥99	≥99	≥99
4-TPP/COU/B-CD	21.3 ± 3.1	36.7 ± 2.0	44.1 ± 1.1	51.6 ± 0.2	57.3 ± 1.2
4-TPP/CIN/B-CD	10.1 ± 1.5	12.1 ± 3.4	6.7 ± 0.8	12.7 ± 0.3	13.4 ± 1.9

**Table 4:** Results for DPPH scavenging assay, comparing the antioxidant on its own, conjugated with 4-TPP and the inclusion complex with β-cyclodextrin.

DPPH Radical Scavenging Activity (%)					
Sample Concentration (mM)					
Sample Name	0.01	0.05	0.1	0.25	0.5
CAF	27.8 ± 9.2	68.9 ± 1.1	90.2 ± 3.8	95.5 ± 0.2	95.5 ± 0.1
COU	47.6 ± 1.4	54.25 ± 1.0	60.9 ± 0.6	65.2 ± 3.0	67.7 ± 1.6
CIN	8.6 ± 0.7	6.6 ± 0.8	7.3 ± 0.5	6.2 ± 1.3	8.1 ± 0.7
4-TPP/CAF	58.4 ± 1.9	95.4 ± 0.2	95.7 ± 0.1	95.6 ± 0.1	95.7 ± 0.2
4-TPP/COU	12.2 ± 1.8	13.1 ± 1.2	19.1 ± 3.1	38.1 ± 3.7	65.2 ± 2.1
4-TPP/CIN	9.6 ± 1.5	7.9 ± 1.7	8.4 ± 1.7	8.5 ± 2.1	10.6 ± 0.9
4-TPP/CAF/B-CD	31.1 ± 2.0	92.3 ± 1.7	95.3 ± 0.1	95.4 ± 0.1	95.2 ± 0.2
4-TPP/COU/B-CD	11.9 ± 0.8	18.4 ± 1.4	28.6 ± 1.6	41.2 ± 7.8	62.3 ± 6.5
4-TPP/CIN/B-CD	10.8 ± 2.5	10.2 ± 0.9	9.6 ± 3.5	9.1 ± 0.8	11.5 ± 1.3

**Table 5:** Calculated HOMO and LUMO energies, electronegativity (*χ*), global hardness (*η*), global electrophilicity index (*ω*), ionization potential (*I*), and electron affinity (*A*) (in eV) of caffeic acid, *ρ*-coumaric acid, cinnamic acid, Compound **2**, Compound **3**, Compound **4**, and TPP.

Compound	ϵ _HOMO_	ϵ	ΔE_gap_	χ	η	ω	I	A
Caffeic acid	−7.37	−1.16	6.21	4.26	3.10	2.93	7.37	1.16
ρ-coumaric acid	−7.50	−1.15	6.35	4.32	3.18	2.94	7.50	1.15
Cinnamic acid	−7.98	−1.27	6.71	4.63	3.35	3.19	7.98	1.27
Compound **2**	−7.26	−0.90	6.36	4.08	3.18	2.62	7.26	0.90
Compound **3**	−7.39	−0.88	6.51	4.13	3.25	2.63	7.39	0.88
Compound **4**	−7.83	−1.03	6.80	4.43	3.40	2.89	7.83	1.03
TPP	−8.68	−0.81	7.87	4.74	3.93	2.86	8.68	0.81

## Data Availability

Data presented in this study are available in the article and Supplementary Materials.

## Supplementary Materials

The following supporting information can be downloaded at: https://www.mdpi.com/article/10.3390/antiox14040465/s1, Figure S1: 1H-NMR and 13C-NMR spectra for compound **2a**; Figure S2: 1H-NMR and 13C-NMR spectra for compound **2b**; Figure S3: 1H-NMR and 13C-NMR spectra for compound **2c**; Figure S4: Molecular Electrostatic Potential maps (in a.u.) and graphical representation of the radical *f*^0^ Fukui function of Compound **2**, Compound **3**, and Compound **4**. All isosurfaces for Fukui functions were generated at a 0.0025 a.u. at the M06-2X-D3/6-311++G(d,p) level of theory; Figure S5: RMSD fluctuation of tight-binding molecular dynamics at 300 K for different orientations (A and B) for TPP antioxidants in the structure *β*-CD inclusion complexes.
