# Supplementary material for: Exploring the Mechanism of β-Cyclodextrin-Encased Phenolic Acids Functionalized with TPP for Antioxidant Activity and Targeting
Source: Antioxidants (Basel). 2025 Apr 13;14(4):465. doi: 10.3390/antiox14040465 (PMC12023939; doi:10.3390/antiox14040465)
Supplement: Supplementary file 1 [file antioxidants-14-00465-s001.zip › antioxidants-3544819-supplementary.pdf]

Supplementary Information.

Exploring the Mechanism of β-Cyclodextrin-Encased Phenolic Acids Functionalized with TPP for Antioxidant Activity and Targeting

Christopher Sbarbaro [1,†], Valeria Márquez-Miranda [1,†], Matías Leal [2], Ricardo Pino-Rios [3], Pedro Olivares [1], Makarena González [1], Ignacio Díaz-Franulic [1], Fernando González-Nilo [1], Osvaldo Yáñez [4] and Yorley Duarte [1,*]

[1] Center for Bioinformatics and Integrative Biology, Facultad de Ciencias de la Vida, Universidad Andrés Bello, Santiago 8370035, Chile
[2] Departamento de Química Orgánica y Fisicoquímica, Facultad de Ciencias Químicas y Farmacéuticas, Universidad de Chile, Santiago 8380494, Chile
[3] Instituto de Ciencias Exactas y Naturales (ICEN), Universidad Arturo Prat, Playa Brava 3256, Iquique 1111346, Chile
[4] Centro de Modelación Ambiental y Dinámica de Sistemas (CEMADIS), Universidad de las Américas, Santiago 7500975, Chile
* Correspondence: yorley.duarte@unab.cl
† These authors contributed equally to this work.

Chemistry

NMR characterization of synthetized molecules

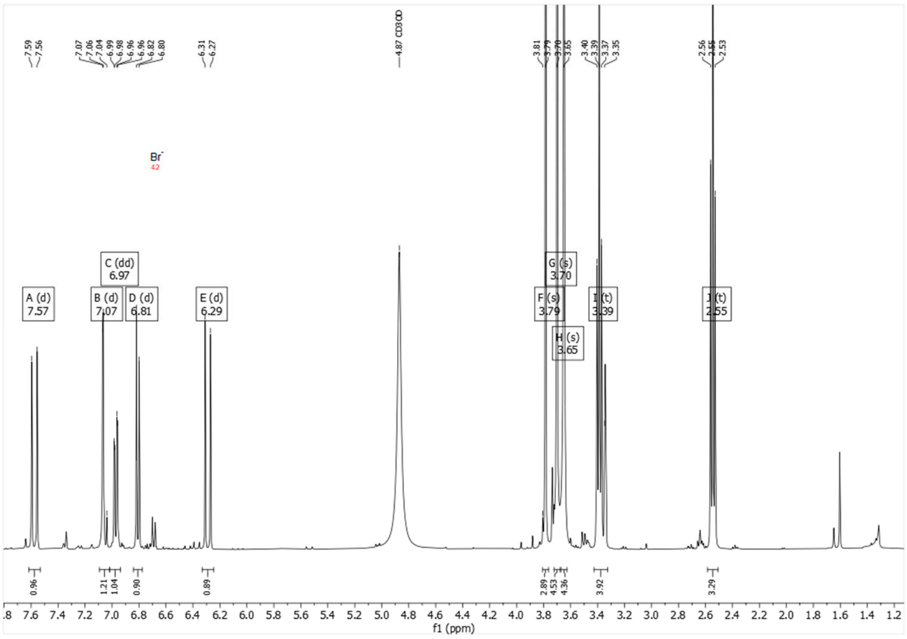

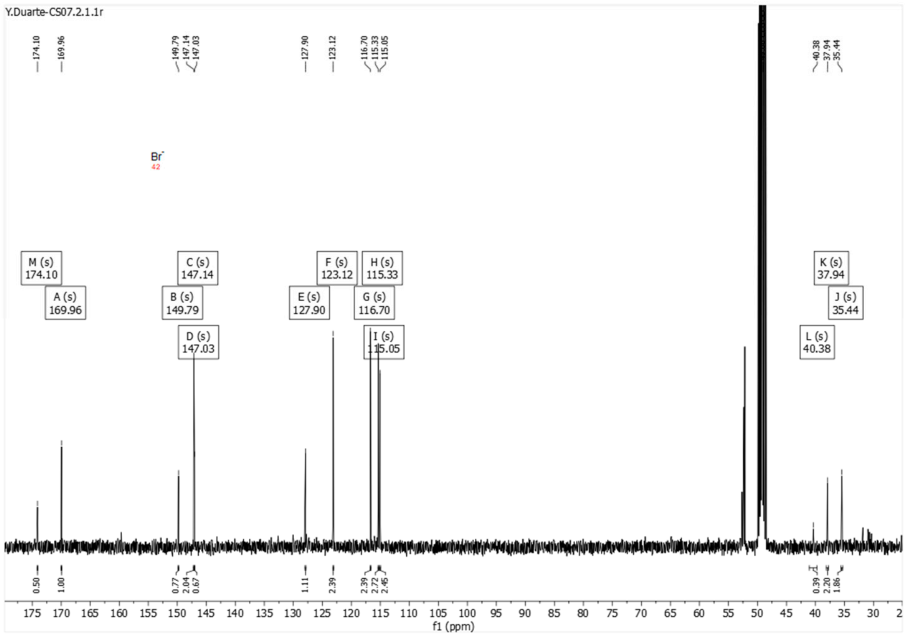

Supplementary Figure 1. 1H-NMR and 13C-NMR spectra for compound 2a.

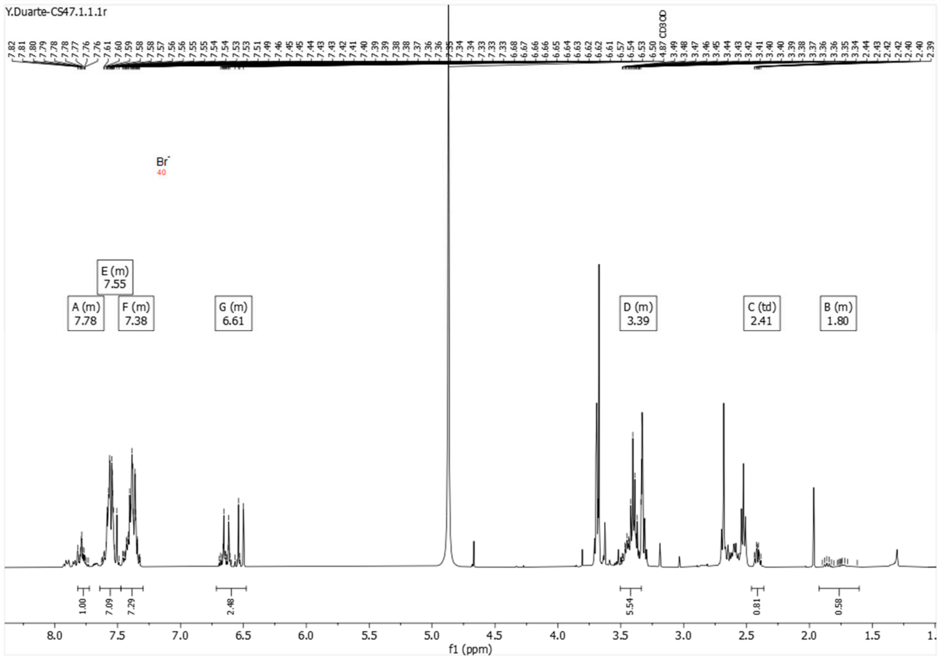

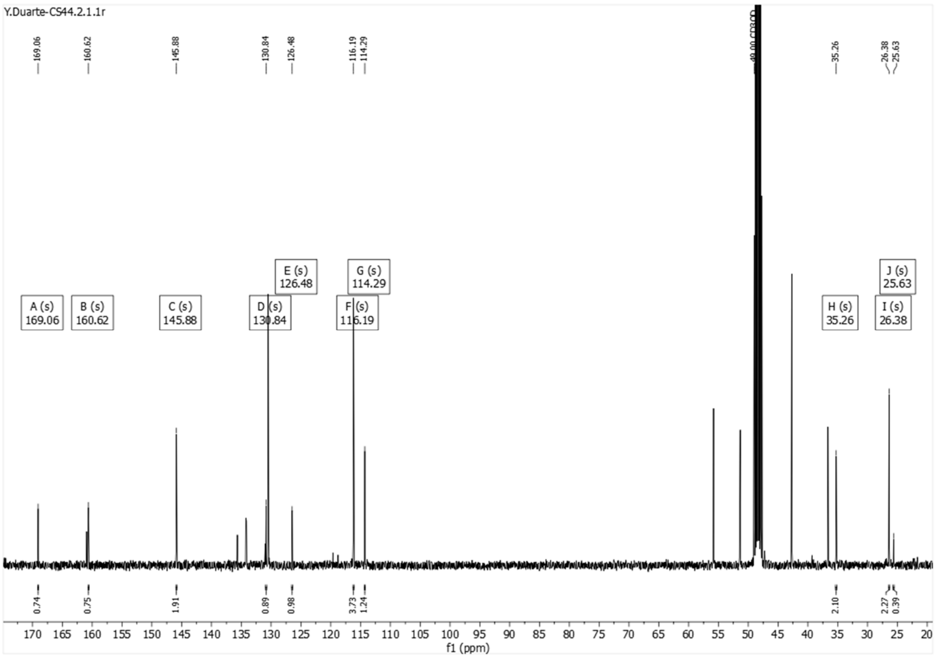

Supplementary Figure 2. 1H-NMR and 13C-NMR spectra for compound 2b.

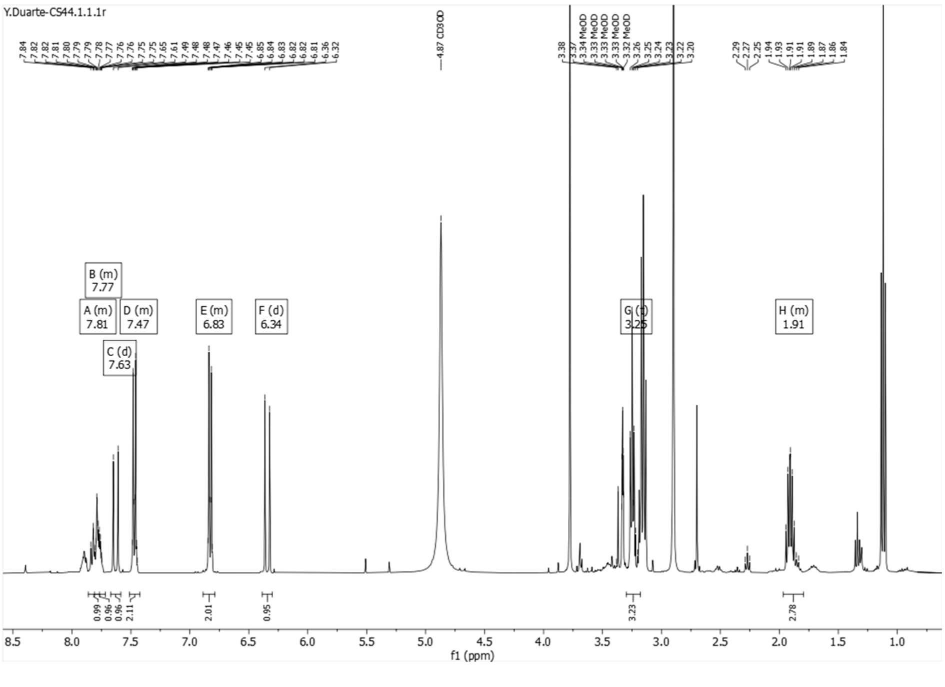

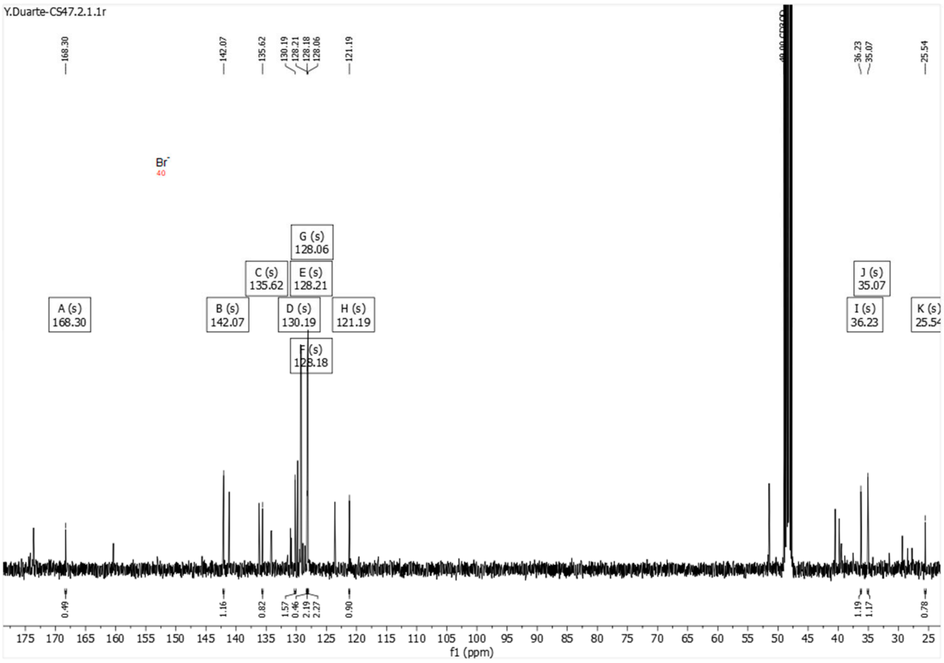

Supplementary Figure 3. 1H-NMR and 13C-NMR spectra for compound 2c.

Molecular Reactivity Analysis

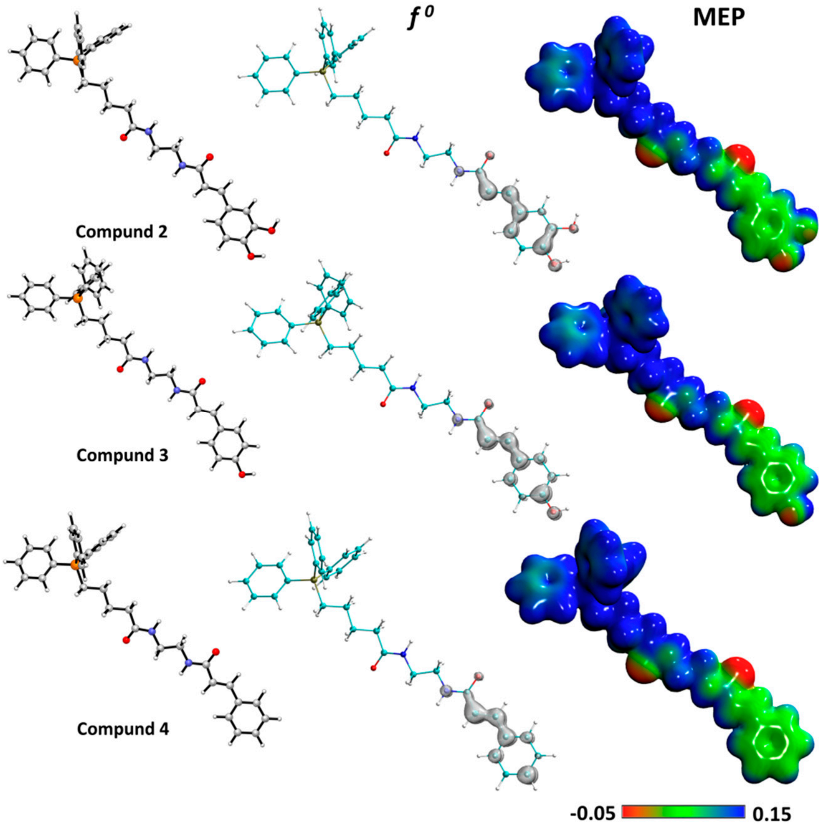

Supplementary Figure 4. Molecular Electrostatic Potential maps (in a.u.) and graphical representation of the radical f^0 Fukui function of Compound 2, Compound 3 and Compound 4. All isosurfaces for Fukui functions were generated at a 0.0025 a.u. at the M06-2X-D3/6-311++G(d,p) level of theory.

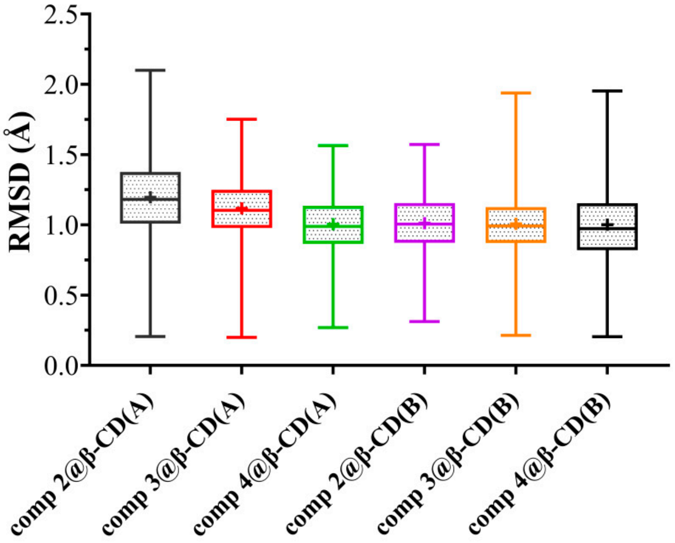

Supplementary Figure 5. RMSD fluctuation of tight-binding molecular dynamics at 300 K for different orientations (A and B) for TPP antioxidants in the structure β-CD inclusion complexes.
